# Expected vs. real growth of companies listed on the London Stock Exchange

**DOI:** 10.1007/s12197-023-09623-0

**Published:** 2023-03-25

**Authors:** Piotr Pietraszewski, Agata Gniadkowska-Szymańska, Monika Bolek

**Affiliations:** 1 Faculty of Economics and Sociology, Department of Capital Market and Investments, University of Lodz, Lodz, Poland; 2 Faculty of Economics and Sociology, Department of Corporate Finance, University of Lodz, Lodz, Poland

**Keywords:** Growth opportunity, Growth of companies, Small and mature companies, **G32**

## Abstract

The aim of this paper is related to the growth opportunity measures representing the expected growth and their relationship with the real growth of companies. The motivation for this research is based on the evidence that growth opportunity measures may fail to predict future growth as measured by earnings per share. In the presented paper the companies listed on LSE are divided for two groups depending on the market they are traded: FTSE100 index and AIM all-share index. It was found that in both samples the growth opportunity measures relationship with the real growth is significant. The difference between companies listed on the main and alternative markets may influence the efficiency of growth opportunity measures predicting the future growth, and therefore the samples should be analyzed separately. The methods applied in this paper include the correlation analysis between growth opportunity and real growth variables, panel data OLS models with fixed effects and the differences between the correlation and regression tests. It can be concluded that growth opportunity measures predict the growth of companies as measured by EPS.

## Introduction

The expected growth included in share pices and the real growth of companies in a capital market determine each other. Investors assess companies based on their potential to increase the market value of their shares. If investors decide to invest capital on the stock exchange, such a decision is linked to the expected rate of return, which is consequently related to the company’s growth.The growth of enterprises and the expectations related to it are part of the valuation of shares carried out by investors in the investment process (Dechow et al. 2000).

The crucial issue that should be considered in relation to the company’s growth is how it is measured. The most popular growth indicators are assets, equity, sales, employment, and earnings per share. Earnings per share (EPS) is a measure directly related to the growth of value, and it is the most important measure in the expected growth analysis (Danbolt et al. 2011). Growth as measured by earnings per share EPS is related directly to the increase in fundamental value, which should be reflected in the increase in the share price. For this reason, apart from the increase in assets, equity and sales, the increase in earnings per share can be considered the most important factor affecting the value of an economic unit. Investors are willing to invest capital when they believe that a company’s value and its shares’ price will increase, so their decisions are reflected in stock prices and are related to measures of growth opportunities (Foucault and Gehrig 2008). This is why it is expected that growth opportunity measures calculated based on the share price, should be related to a company’s real growth.

The study aims to analyze the relationship between the measures of growth opportunity and the future growth of companies listed on the London Stock Exchange main market represented by enterprices included in FTSE100 (Financial Times Stock Exchange 100) index and the AIM (Alternative Investment Market) alternative exchange traded companies. The FTSE100 index, is a share index of the 100 companies listed on the London Stock Exchange with the highest market capitalization, many of them are internationally focused corporations. The main market of LSE is dedicated for more established companies (mid- and large-cap businesses) from across all sectors. Meanwhile, AIM provides growing companies with access to a set of institutional and retail investors. The AIM is a sub-market of the LSE that is designed to help smaller companies access capital from the public market. AIM allows these companies to raise capital by listing on a public exchange with much greater regulatory flexibility compared to the main LSE stock market^1^.

In this research paper the following hypothesis are tested: (1) the growth opportunity ratios are significantly related to the future real growth of enterprises listed on LSE and (2) there are significant differences between tested samples and they may explain earlier failures in finding the relationship between growth opportunity and the real growth of companies as presented by Danbolt et al. (DHJ) (2011). Simillar analysis for Polish main and alternative markets and companies listed on the Warsaw Stock Exchange was presented by Bolek et al. (2021). The motivation of this paper is related to the application of the presented method on a more developed UK market. To test the hypothesis, the growth indicators and growth opportunity ratios are calculated and correlation between variables, panel data OLS models are analyzed, and the differences between the samples are tested.

The paper comprises the following sections: first, an overview of the literature and the growth opportunity measures are presented. Then the data and methods are overviewed, followed by the results, a discussion, and conclusions.

## Literature overview

A company’s value can be described not only in terms of different standards, but also in terms of the factors that create it, namely value drivers (Richards and Jones 2008; Spieth et al. 2019; Marr 2007). Value drivers can be identified as indicators that are characteristic of financial analysis (Marr 2008). Company growth is usually associated with the value and the global profit generated each year, EPS, sales, an increase in the size of the enterprise, and improved labor productivity and employment rates (Barringer et al. 2005; Tomisawa and Hashimoto 2008). The increase in assets, equity, sales, and EPS should be reflected in the growth of the value of the enterprise if it implements profitable investment projects (Seelos and Mair 2007). The increase in the fundamental value of a company is directly related to the price of its shares and effective capital market mechanisms (Pfitzer et al. 2013; Kutsenko and Smyrnov 2019; Gusov et al. 2011; Revutskii 2014). An enterprise’s value is largely determined by the value of its resources, measured by the usefulness of its products and the efficiency of its sales (Neely 2002). The more unique resources that a company possesses, the greater its value. Nowadays, the traditional concept of value has been partially replaced by intellectual capital, which is often regarded as a competitive advantage (Sardo and Serrasqueiro 2017; Moon and Kym 2006; Setiany et al. 2020; Ting et al. 2020; Nguyen and Doan 2020; Salvi et al. 2020).

Companies’ operating decisions are related to the products they offer and their prices, as well as operating costs, and distribution, considering the preferences of buyers and competitors (López Salazar et al. 2012). The result of these decisions is the growing dynamics of sales and operating profit margin, as well as profitability, although earnings are also affected by the tax burden. Investment decisions depend on the size and structure of fixed assets and net working capital. On the other hand, financial decisions related to the structure of invested capital affect the risk of the business and determine the weighted average cost of capital. To sum up, an enterprise should act in such a way that meets investors’ expectations (Mao 2009; Hung and Liu 2009; Avlasenko et al. 2020).

Company growth that is related to assets, equity, sales, and EPS should result in an increase in its value. Companies raise capital based on a valuation carried out by investors. The higher the value of the enterprise, which reflects its growth potential, the more capital the company can raise on the primary market. Valuation is, therefore, a key aspect in assessing growth (Barkham and Ward 1999; Pernamasari 2020; Ignatyuk et al. 2021).

Investors on a capital market directly or indirectly transfer their accumulated capital to companies, and in return, they expect a positive rate of return that covers the risk associated with the investment (Beck and Dermirguc-Kunt 2006; Levine et al. 2000). Earnings growth increases the enterprise’s intrinsic value, the market price of its shares, and ultimately the rate of return and its market value (Balzer et al. 2020). The whole perspective related to capital market mechanisms allows us to understand earnings growth from the point of view of investors, whose expectations are related to rates of return. Therefore, the role of capital market institutions in developing an enterprise is crucial, mainly due to the maximization of its profitability and access to capital (Greenwood and Jovanovic 1990). A company characterized by growth potential can easily raise capital for further development.

Depending on the definition, an enterprise’s value can be determined in various ways. Modigliani and Miller (1961) considered tax savings and the costs of potential financial distress, concluding that there is an optimal amount of debt for every enterprise (Czerwonka and Jaworski 2021). Thus, it can be concluded that an enterprise’s value is determined by future income streams, which are closely related to the enterprise’s asset management and financial structure (Kishibayeva et al. 2020; Benninga and Sarig 1997) proposed measuring the value of an enterprise as the sum of the value of equity, debt, and all other securities that do not belong to working capital.

Growth opportunity, as a proxy, was considered by Rajan and Zingales (1995), Michaelas et al. (1999), Ozkan (2001), and Billett et al. (2007) in the light of an optimal capital structure that influences a company’s value and growth. Martinez-Sola et al. (2018) found that SMEs with greater growth opportunities adjust more quickly to their target cash holding level to preserve their financial flexibility and to take advantage of profitable investment opportunities when they grow. On the other hand, improving supply chain management positively influences company development (Wahyuni and Sumarmi 2018). Firms with high growth potential face high risk and adopt a more progressive strategy for earnings (Huang et al. 2018). Growth opportunity is also created by the institutional investors that support the development of enterprises, and as Urbano et al. (2019) stated, it could be possible to obtain economic growth by encouraging the appropriate institutions to increase entrepreneurship.

Tobin (1969) presented an index of the market value of assets and their replacement costs, which is considered a growth opportunity measure. Due to the problems associated with determining the level of replacement costs, it is possible to modify the Tobin’s Q ratio in line with DHJ proposal. The Kester (1984) and Brealey and Myers (1981) model (KBM) was built based on the decomposition of the stock price into the value of assets in place and the value of the growth opportunity. Additional proposals for measuring growth opportunity, presented by Otto (2000), are related to the concept of value added.

Growth patterns may be different in separate stages of a company’s development, especially in a capital market, where exchanges provide specific requirements for companies planning to issue shares. This paper proposes a modified approach related to dividing the companies based on the market where they issue their shares. This approach may help solve companies’ growth inconsistence (“puzzle”), described by DHJ, who found that growth opportunity measures did not affect companies’ growth as reflected by EPS growth. On the other hand, Bolek et al. (2021) found a significant impact of expected growth on the future growth of companies listed on the Warsaw Stock Exchange. They analyzed separately a sample of companies that traded on the main and alternative markets and concluded that growth patterns are different in companies characterized by different stages of development.

## Data and methods

This article examines companies listed on the London Stock Exchange (LSE) that were included in the FTSE100 index and the AIM all-share index. The data originate from the Bloomberg database. The analysis was carried out on data covering the periods 1971–2018 for the FTSE100 and 1980–2018 for AIM (up to the beginning of the COVID-19 pandemic). The share prices were adjusted for capital changes in the type of subscription rights, dividends, and splits.

The database contained 2584 observations (company/year) for the FTSE100 companies and 1794 records for the AIM companies and their growth ratios. However, these databases did not allow us to calculate the growth opportunity indicators for all company/year observations due to the lack of required information.

In this paper, company growth is represented by the growth of assets, equity, sales, and EPS. The one-, three-, five-, eight- and ten-year growth rates of assets, equity, and sales are calculated according to the following formula:

1 $$\varDelta {X}_{+n}=\frac{{X}_{n}-{X}_{0}}{{X}_{0}},$$

where *n* = 1, 3, 5, 8, 10, accordingly, and X_n_ denotes total assets (TA), equity (E ), or sales (S) at the end of *n* years after the year in which total earnings equals X_0_.

The growth rates of earnings per share are determined as follows:

2 $$\varDelta EP{S}_{+n}=\frac{EP{S}_{n}-EP{S}_{0}}{{TA}_{0}},$$

where: EPS_n_ is earnings per share in *n* years after year 0. Earnings growth is calculated in relation to asset size (TA) since earnings can be negative and affect the results.

Next, the methods of calculating growth oppotrunity indicators are presented. All measures of growth opportunity are based on the idea that the market prices of shares reflect a company’s future growth prospects (investors’ expectations).

Tobin (1969) presented an index of the market value of assets and their replacement costs, which is considered a growth opportunity measure. The value of the TQ ratio was taken directly from Bloomberg Database (Q1). Due to the problems associated with determining the level of replacement costs in Tobin’s fotmula (Tobin 1969), it is possible to modify the Tobin’s Q ratio in line with DHJ proposal (Q2):

3 $$TQ=\frac{TA+MVE-BVE}{TA}$$

where: TA – total assets, MVE – market value of equity, BVE – book value of equity.

The higher the value of this index, the greater the enterprise’s growth opportunity based on the assumption that the difference between the market value of equity and its book value determines the growth potential, which is included in the market price of shares.

The Kester (1984) and Brealey and Myers (1981) model (KBM) was built based on the decomposition of the stock price into the value of assets in place and the value of the growth opportunity.

4 $${P}_{g}KBM=\frac{{P}_{S}-EPS/{k}_{e}}{{P}_{S}}$$

where: P_g_ – value of growth potential, P_s_ – share price, EPS – earnings per share, k_e_ – cost of equity.

The higher the value of this indicator, the greater the growth opportunity, as reflected by the market. This model should not be used when the company’s profits are negative.

The next proposals for measuring growth opportunity are presented by Otto (2000), and are related to the concept of value added. The first model refers to the value that exceeds the company’s value (EVF – Exceeding Value to Firm):

5 $${P}_{g}EVF=\frac{(MVE+BVD)-(BVE+BVD)}{MVE+BVD}.$$

where: MVE – market value of equity, BVE – book value of equity, BVD – book value of debt.

The second model represents a value that exceeds the shareholders’ value (EVE – Exceeding Value to Equity):

6 $${P}_{g}EVE=\frac{MVE-BVE}{MVE},$$

The higher the value of the growth opportunity indicators, the greater the expected growth of the enterprise reflected in the share prices.

The group of the capital market ratios and their levels can be explained in relation to the growth potential. First indicator that can be used to assess the growth opportunity of an enterprise is the P/E price-earnings ratio, also used in its inverse form to avoid the situation when EPS is equal to zero. The higher the P/E value, the greater the company’s growth potential. Similarly, the MV/BV market to book value ratio shows how many times the market price of a share exceeds its book value, which can be interpreted as an additional expected value in relation to the book value of shares.

After calculating the growth indices and growth opportunity ratios, the correlation coefficients between the levels of various growth opportunity measures and future company growth are calculated. This relationship is also studied more deeply using the multivariate regression model according to the formula proposed by DHJ. Besides growth opportunity measures, the model includes other factors associated with EPS growth identified in the literature. In each estimated linear regression, one of the growth opportunity measures is only one of several explanatory variables. Therefore, these estimations allow us to explore whether the level of growth opportunities has any incremental impact on earnings growth once a control for other factors potentially related to that growth is considered.

The presence of fixed effects was tested with the Wald test. The fixed effects were analyzed both for the objects (companies) and for the years. Correction for heteroscedasticity was applied each time it appeared in regression. In the joined version of the test, where two types of fixed effects were considered simultaneously, the panel regression was reflected by the following Eq. (7):

7 $$gEP{S}_{it}={\alpha }_{i}+{\gamma }_{t}+{\beta }_{1}G{O}_{0it}+{\beta }_{2}RO{E}_{-1it}+{\beta }_{3}\varDelta EP{S}_{0it}+{\beta }_{4}\varDelta T{A}_{0it}+{\beta }_{5}{ln\;}M{\;V}_{0it}+{\varepsilon }_{it}$$

In the above Eq. (7), $gEPS$ refers to the one-year, two-year or three-year growth of EPS. $RO{E}_{-1}=EP{S}_{-1}/E{Q}_{-1}$ denotes a one-year-lagged return on equity, $\varDelta EP{S}_{0}=\frac{EP{S}_{0}-EP{S}_{-1}}{TA{S}_{-1}}$, $\varDelta T{A}_{0}=\frac{{TA}_{0}-{TA}_{-1}}{{TA}_{-1}}$, $ln\;M\;V_0$ is the natural logarithm of the market value and $G{O}_{0}$ represents one of growth opportunity measures included in the analyzis.

The one-year-lagged return on equity ($RO{E}_{-1}$) is included in the regression to cover the effect of mean reversion in earnings. Mean reversion is observed when coefficient ${\beta }_{2}$ is negative and statistically significant. The recent one-year earnings growth, $\varDelta EP{S}_{0}$, is added to control for the persistence in earnings growth rates (when ${\beta }_{3}$ is positive). However, to some extent, both control variables embody similar information, and each of them can speak for either the mean reversion or the persistence in earnings, depending on whether the sign of the respective regression coefficient is positive or negative. The presence of recent annual growth of total assets $\varDelta T{A}_{0}$ is slightly more arbitrary and based on its strong predictive power for future abnormal returns observed in the literature. Finally, the present market value logarithm, *lnMV*, is a proxy for company size. All constants ${\alpha }_{i}$ have the same value for all objects: ${\alpha }_{i}={\alpha }_{0}=const$ and all constants ${\gamma }_{i}$ have the same value for all years: ${\gamma }_{t}={\gamma }_{0}=const$. The *F* statistic, given by the following formula, is used to verify the hypothesis:

8 $$F=\frac{\left({RSS}_{OLS}-{RSS}_{FE}\right)/\left(N-1\right)}{{RSS}_{FE}/\left(NT-N-K\right)}$$

Where ${SSR}_{OLS}$ is the residual sums of squares in the pooled OLS regression, ${SSR}_{FE}$ is the residual sums of squares in the regression with fixed effects, *N* is the number of objects, *T* is the number of periods, and *K* = 5 stands for the number of explanatory variables.

To verify the significance of the difference between the two Pearson correlation coefficients from the two independent populations, a t-test based on the Fisher transformation is used. The null hypothesis states that these correlation coefficients are equal. The t-statistic is given by the following formula:

9 $$t=\frac{{z}_{FTSE}-{z}_{AIM}}{\sqrt{\frac{1}{{n}_{FTSE}-3}+\frac{1}{{n}_{AIM}-3}}},$$

where ${z}_{FTSE}=\frac{1}{2}{ln}\left(\frac{1+{r}_{FTSE}}{1-{r}_{FTSE}}\right)$, ${z}_{AIM}=\frac{1}{2}{ln}\left(\frac{1+{r}_{AIM}}{1-{r}_{AIM}}\right)$, ${r}_{FTSE}$, ${r}_{AIM}$ denote the estimated correlation coefficients in the two samples of companies, and ${n}_{FTSE},{n}_{AIM}>3$ are the numbers of companies in each sample. If ${r}_{FTSE}$ is greater than ${r}_{AIM}$, the resulting value of *t* will have a positive sign; if it is smaller, the sign will be negative. The test statistic has a t-Student distribution with ${n}_{FTSE}+{n}_{AIM}-4$ degrees of freedom.

Two tests for a structural break in regression coefficients are applied in the next step to verify the differences between surveyed groups of companies. The first one, the Chow test, is based on the F statistic and allows us to compare the residual sums of squares in two regressions: one estimated for the joint group of companies:

10 $${EPSGrowt}_{i}=\alpha +{\beta }_{1}G{O}_{0i}+{\beta }_{2}RO{E}_{-1i}+{\beta }_{3}\varDelta EP{S}_{0i}+{\beta }_{4}\varDelta T{A}_{0i}+{\beta }_{5}{ln}M{V}_{0i}+{\varepsilon }_{i},$$

and an auxiliary regression:

11 $${EPSGrowt}_{i}=\alpha +{\beta }_{1}G{O}_{0i}+{\beta }_{2}RO{E}_{-1i}+{\beta }_{3}\varDelta EP{S}_{0i}+{\beta }_{4}\varDelta T{A}_{0i}+{\beta }_{5}{ln}M{V}_{0i}+ \;\;+{\gamma }_{0}{Z}_{i}+{\gamma }_{1}{Z}_{i}G{O}_{0i}+{\gamma }_{2}{Z}_{i}RO{E}_{-1i}+{\gamma }_{3}{Z}_{i}\varDelta EP{S}_{0i}+{\gamma }_{4}{Z}_{i}\varDelta T{A}_{0i}+{\gamma }_{5}{Z}_{i}{ln}M{V}_{0i}+{\varepsilon }_{i},$$

where *Z*_*t*_ is a dummy variable equal to 1 for the FTSE companies and 0 for the AIM companies (or vice versa).

The joint null hypothesis, ${H}_{o}{:\gamma }_{i}=0$ for $i=0, \dots ,5$, is tested against the alternative hypothesis that one or more of the restrictions under ${H}_{o}$ does not hold. The *F* statistic, given by the formula:

12 $$F=\frac{\left(RS{S}_{R}-RS{S}_{UR}\right)/q}{RS{S}_{UR}/(N-k-1)}=\frac{\left(RS{S}_{R}-RS{S}_{UR}\right)/6}{RS{S}_{UR}/(N-12)},$$

where RSS_R_ and RSS_UR_ denote residual sums of squares in the restricted and unrestricted models, *N* is the number of observations, and *k* is the number of regression coefficients (intercept not included) in the unrestricted model, has the ${F}_{6,N-12}$ distribution.

In the likelihood ratio test, the values of the likelihood functions of both Eqs. (10) and (11), ${L}_{R},{L}_{U}$, respectively, are compared. The probability distribution of the log-likelihood ratio statistic, given by formula $-2{ln}({L}_{R}/{L}_{U})$, is approximately a chi-squared distribution with degrees of freedom equal to the number of restrictions, *q* = 6.

The following research hypothesis are tested:

1. There is a significant relationship between growth opportunity measures and real growth in companies listed on LSE main (FTSE100 Index) and alternative (AIM) markets,
2. There are significant differences between the surveyed groups of companies and it is necessary to analyze them separately.

## Results

In this section, the correlation and regression parameters results are presented first for the FTSE100 index included companies, then the AIM index incluede firms. The differences between the results in both groups of companies are analyzed at the end of this section.

### FTSE100 companies

The expectation of growth should be reflected by different measures, no matter which growth opportunity indicator is applied. It is important for this research to find out the extent to which various measures of growth opportunity can predict (influence) companies’ future growth. The Pearson correlation coefficients between various measures of growth opportunity and the subsequent growth rates of companies are presented in Table 1.

**Table 1:** Growth opportunities and realized growth (FTSE100) – correlation coefficients

	gTA1	gTA3	gTA5	gTA8	gTA10
Q1	0.205^***^	0.237^***^	0.254^***^	0.272^***^	0.271^***^
Q2	0.186^***^	0.218^***^	0.256^***^	0.278^***^	0.265^***^
P/E	0.006	0.002	0.015	0.042^*^	0.043
MV/BV	0.137^***^	0.152^***^	0.201^***^	0.161^***^	0.174^***^
KBM	0.027	-0.043	-0.030	0.002	0.017
EVF	0.202^***^	0.230^***^	0.227^***^	0.225^***^	0.224^***^
EVE	0.160^***^	0.181^***^	0.175^***^	0.153^***^	0.147^***^
	gS1	gS3	gS5	gS8	gS10
Q1	0.147^***^	0.206^***^	0.226^***^	0.245^***^	0.212^***^
Q2	0.124^***^	0.198^***^	0.209^***^	0.234^***^	0.218^***^
P/E	0.020	0.051^**^	0.055^**^	0.107^***^	0.113^***^
MV/BV	0.103^***^	0.128^***^	0.153^***^	0.158^***^	0.139^***^
KBM	0.042	0.022	0.023	0.047	0.029
EVF	0.132^***^	0.177^***^	0.187^***^	0.206^***^	0.212^***^
EVE	0.102^***^	0.123^***^	0.133^***^	0.138^***^	0.160^***^
	gE1	gE3	gE5	gE8	gE10
Q1	0.129^***^	0.150^***^	0.165^***^	0.124^***^	0.133^***^
Q2	0.090^***^	0.139^***^	0.160^***^	0.120^***^	0.124^***^
P/E	-0.042^*^	-0.001	0.081^***^	0.064^**^	0.085^***^
MV/BV	0.177^***^	0.221^***^	0.178^***^	0.150^***^	0.194^***^
KBM	0.035	-0.004	0.032	0.040	0.057^*^
EVF	0.162^***^	0.183^***^	0.177^***^	0.142^***^	0.143^***^
EVE	0.174^***^	0.201^***^	0.192^***^	0.164^***^	0.161^***^
	gEPS1	gEPS3	gEPS5	gEPS8	gEPS10
Q1	0.172^***^	0.223^***^	0.215^***^	0.276^***^	0.254^***^
Q2	0.140^***^	0.202^***^	0.191^***^	0.252^***^	0.239^***^
P/E	0.062^***^	0.062^***^	0.044^*^	0.044^*^	0.025
MV/BV	0.118^***^	0.173^***^	0.159^***^	0.178^***^	0.155^***^
KBM	0.123^***^	0.087^***^	0.059^**^	0.105^***^	0.093^***^
EVF	0.156^***^	0.174^***^	0.155^***^	0.212^***^	0.205^***^
EVE	0.143^***^	0.153^***^	0.111^***^	0.154^***^	0.148^***^

Note: Shaded cells indicate that the coefficient is significant and of the predicted sign

∗/∗∗/∗∗∗ The coefficients are significant at the 10% / 5% / 1% level

The future growth in total assets is predicted by all market-to-book-based growth opportunity measures (Tobin Q, MV/BV, EVF, EVE). For each period, the correlation coefficients are statistically significant at the 1% confidence level and are of the predicted signs. Among these four measures, EVE is the least correlated with asset growth. In contrast, P/E and KBM are poor predictors of future growth in total assets – almost all coefficients are not statistically significant.

Quite a similar pattern of relationships exists between growth opportunity measures and future growth rates when equity and sales growth are concerned; there are only some differences in the strength of these correlations. In the case of earnings growth, price-to-earnings-based measures also play an important role; KBM is statistically significant at the 1% level for each of the five periods taken into consideration, but correlation coefficients are lower than for the market-to-book-based ratios.

A relative change in EPS indicates the growth of a company’s future value, and according to DHJ methodology, there are indicators of the growth that influence it.

In the next step, panel regressions with fixed effects were estimated. The results are presented in Table 2.

**Table 2:** Determinants of future earnings EPS growth (FTSE100) – Fixed-effects

	Sample	Const.	ROE_− 1_	ΔEPS_0_	ΔTA_0_	lnMV_0_	GO_0_	R^2^%	Adj. R^2^%	F-stat. F-stat.
Explained variable: One-year EPS growth										
Q1	2059	0.004^**^	-7e-07	-3.160^***^	-0.0001^*^	-0.0006^***^	0.002^***^	23.1	17.8	4.35^***^
Q2	2060	0.0005	-7e-07	-3.433^***^	-9e-05	-5e-05	0.0009^***^	21.8	16.4	4.05^***^
P/E	2063	0.0008	4e-07	-2.491^**^	-0.0001^*^	-4e-05	3e-05^***^	19.0	15.5	5.39^***^
MV/BV	2054	0.003	-9e-07	-2.789^***^	-0.0001	-0.0002	0.0003^***^	20.8	15.3	3.81^***^
KBM	1557	-0.117^***^	1e-07	-2.399^**^	-0.0001^*^	-0.0006^*^	0.115^***^	29.6	23.8	5.12^***^
EVF	2059	0.008^***^	-8e-07	-3.226^***^	-0.0001	-0.0009^***^	0.006^***^	23.2	17.9	4.37^***^
EVE	2050	0.006^***^	2e-08	-2.333^**^	-0.0001^*^	-0.0006^**^	0.004^***^	25.7	20.5	4.94^***^
Explained variable: Three-year EPS growth										
Q1	1910	0.033^***^	6e-06	-8.804^***^	-0.0002	-0.004^***^	0.004^***^	42.5	38.2	10.0^***^
Q2	1918	0.009^***^	1e-05^***^	-7.639^***^	-0.0001	-0.009^***^	0.002^***^	41.1	36.8	9.51^***^
P/E	1917	0.008^***^	3e-05^***^	-2.735	-0.0002	-0.0006^**^	0.0001^***^	42.1	37.9	9.98^***^
MV/BV	1906	0.028^***^	-2e-05^*^	-10.56^***^	-9e-05	-0.003^***^	0.001^***^	41.6	37.3	9.72^***^
KBM	1403	-0.279^***^	2e-05^**^	2.611	-0.0002	-0.002^***^	0.303^***^	49.0	44.4	10.6^***^
EVF	1910	0.041^***^	9e-06	-9.131^***^	-0.0001	-0.005^***^	0.017^***^	42.6	38.4	10.1^***^
EVE	1894	0.036^***^	1e-05^**^	-4.592^**^	-0.0002	-0.004^***^	0.008^***^	43.5	39.3	10.3^***^
Explained variable: Five-year EPS growth										
Q1	1756	0.077^***^	7e-06	-19.12^***^	-0.0001	-0.009^***^	0.006^***^	54.6	51.0	15.2^***^
Q2	1767	0.023^***^	2e-05	-18.86^***^	-0.0002	-0.002^***^	0.004^***^	51.9	48.1	13.7^***^
P/E	1757	0.019^***^	6e-05^***^	-11.76^***^	-0.0002	-0.002^***^	0.0001^***^	51.6	47.8	13.7^***^
MV/BV	1756	0.067^***^	-2e-05	-18.47^***^	-7e-05	-0.007^***^	0.002^***^	51.3	47.5	13.4^***^
KBM	1253	-0.351^***^	3e-05^**^	-15.98^***^	-0.0002	-0.007^***^	0.423^***^	58.4	54.3	14.03^***^
EVF	1756	0.092^***^	9e-06^***^	-19.88^***^	-0.0001	-0.011^***^	0.027^***^	54.9	51.3	15.36^***^
EVE	1733	0.088^***^	2e-05^**^	-17.39^***^	-0.0002	-0.010^***^	0.013^***^	53.1	49.4	14.2^***^
Explained variable: Ten-year EPS growth										
Q1	1378	0.202^***^	3e-05	-22.17^**^	-0.0004	-0.026^***^	0.018^***^	64.2	60.0	19.5^***^
Q2	1390	0.050^****^	5e-05	-21.73^**^	-0.0005	-0.006^***^	0.011^***^	60.4	56.8	16.7^***^
P/E	1389	0.052^***^	0.0001^***^	-16.54^*^	-0.0007^*^	-0.005^***^	0.0005^***^	59.9	56.3	16.7^***^
MV/BV	1382	0.163^***^	-9e-05^**^	-22.87^***^	-0.0004	-0.018^***^	0.004^***^	59.0	55.3	15.8^***^
KBM	901	-0.486^***^	3e-05^*^	4.290	-0.0007^**^	-0.012^***^	0.615^***^	79.0	76.4	30.4^***^
EVF	1378	0.256^***^	3e-05^**^	-23.12^***^	-0.0002	-0.032^***^	0.082^***^	65.0	61.7	20.2^***^
EVE	1357	0.248^***^	8e-05^***^	-17.13^**^	-0.0007	-0.030^***^	0.039^***^	62.1	58.6	17.7^***^

Note: */**/*** The coefficients or F-statistic are significant at the 10% / 5% / 1% level

The previously presented conclusions about the statistical importance of the incremental impact of growth opportunity measures are still valid and are even more evident. Additionally, EPS growth still proves to be strongly related to firm size, although this effect is less evident in the short, one-year horizon.

### AIM companies

In the second step, the same analyzis as above is repeated for smaller and younger companies traded on the alternative exchange of LSE. The correlation coefficients between growth opportunity measures and the future growth rates for the AIM companies are presented in Table 3.

**Table 3:** Growth opportunities and realized growth (AIM) – correlation coefficients

	gTA1	gTA3	gTA5	gTA8	gTA10
Q1	0.332^***^	0.356^***^	0.386^***^	0.378^***^	0.417^***^
Q2	0.255^***^	0.324^***^	0.319^***^	0.336^***^	0.380^***^
P/E	0.205^***^	0.199^***^	0.213^***^	0.237^***^	0.249^***^
MV/BV	0.330^***^	0.308^***^	0.299^***^	0.269^***^	0.307^***^
KBM	0.071^*^	0.138^***^	0.146^***^	0.094^*^	0.067
EVF	0.294^***^	0.306^***^	0.302^***^	0.274^***^	0.271^***^
EVE	0.242^***^	0.242^***^	0.221^***^	0.166^***^	0.176^***^
	gS1	gS3	gS5	gS8	gS10
Q1	0.243^***^	0.299^***^	0.372^***^	0.348^***^	0.341^***^
Q2	0.190^***^	0.281^***^	0.260^***^	0.294^***^	0.285^***^
P/E	0.223^***^	0.252^***^	0.255^***^	0.281^***^	0.240^***^
MV/BV	0.226^***^	0.235^***^	0.243^***^	0.238^***^	0.220^***^
KBM	0.069^*^	0.126^***^	0.132^***^	0.082^*^	0.052
EVF	0.194^***^	0.235^***^	0.250^***^	0.221^***^	0.181^***^
EVE	0.180^***^	0.183^***^	0.141^***^	0.119^***^	0.087^**^
	gE1	gE3	gE5	gE8	gE10
Q1	0.283^***^	0.313^***^	0.277^***^	0.283^***^	0.287^***^
Q2	0.205^***^	0.318^***^	0.282^***^	0.262^***^	0.321^***^
P/E	0.156^***^	0.172^***^	0.189^***^	0.193^***^	0.235^***^
MV/BV	0.344^***^	0.367^***^	0.284^***^	0.257^***^	0.288^***^
KBM	0.038	0.113^***^	0.144^***^	0.101^**^	0.068
EVF	0.258^***^	0.287^***^	0.235^***^	0.217^***^	0.214^***^
EVE	0.246^***^	0.259^***^	0.182^***^	0.149^***^	0.157^***^
	gEPS1	gEPS3	gEPS5	gEPS8	gEPS10
Q1	0.145^***^	0.140^***^	0.193^***^	0.224^***^	0.249^***^
Q2	0.073^***^	0.117^***^	0.196^***^	0.231^***^	0.220^***^
P/E	0.053^*^	0.077^**^	0.092^***^	0.102^***^	0.077^**^
MV/BV	0.145^***^	0.139^***^	0.153^***^	0.198^***^	0.220^***^
KBM	0.095^**^	0.098^**^	0.114^***^	0.095^*^	0.068
EVF	0.104^***^	0.124^***^	0.152^***^	0.177^***^	0.183^***^
EVE	0.074^***^	0.094^***^	0.109^***^	0.101^***^	0.118^***^

Note: Shaded cells indicate that the coefficient is significant and of the predicted sign

*/**/*** The coefficients are significant at the 10% / 5% / 1% level

Compared with the previous results, growth opportunity measures are as good predictors of future growth for the AIM companies as they were for the FTSE100 companies. For instance, the P/E ratio is now as good a predictor of future growth in total assets, sales, and equity as other market-to-book-based growth opportunity measures (Tobin Q, MV/BV, EVF, EVE). Even KMB performs quite well, especially for future growth in time horizon up to 5 years.

Because the Wald test indicates the presence of fixed effects, panel regressions with fixed effects are presented in Table 4.

**Table 4:** Determinants of future earnings EPS growth (AIM) – Fixed-effects

	Sample	Const.	ROE_− 1_	ΔEPS_0_	ΔTA_0_	lnMV_0_	GO_0_	R^2^%	Adj. R^2^%	F-stat.
Explained variable: One-year EPS growth										
Q1	1311	0.100^***^	−0.0003^*^	0.017	−7e-05	−0.024^***^	0.022^***^	22.2	13.5	2.55^***^
Q2	1328	0.053^**^	-0.0003^*^	0.021	-8e-05	-0.014^***^	0.021^***^	20.7	12.1	2.40^***^
P/E	1216	0.094^***^	0.0001	−0.050	−0.001	−0.017^***^	0.0005^***^	24.1	15.3	2.76^***^
MV/BV	1322	0.128^***^	−0.0003^**^	0.022	−8e-05	−0.030^***^	0.013^***^	22.0	13.4	2.54^***^
KBM	1368	0.159^***^	−0.0002	0.015	-6e-05	−0.033^***^	0.089^***^	21.4	13.0	2.55^***^
EVF	1311	0.153^***^	−0.0003^**^	0.016	−7e-05	−0.034^***^	0.116^***^	22.2	13.5	2.55^***^
EVE	1321	0.163^***^	−0.0003^**^	0.019	−8e-05	−0.032^***^	0.060^***^	20.9	12.1	2.37^***^
Explained variable: Three-year EPS growth										
Q1	1172	0.447^***^	−0.001^***^	0.605^*^	−0.002^**^	−0.099^***^	0.055^***^	40.4	33.2	5.63^***^
Q2	1190	0.344^***^	-0.001^***^	0.580^*^	-0.002^*^	-0.081^***^	0.069^***^	38.1	30.9	5.29^***^
P/E	1086	0.413^***^	−8e-05	−0.242	−0.047^*^	−0.078^***^	0.001^***^	37.9	30.2	4.91^***^
MV/BV	1181	0.566^***^	−0.002^***^	0.578^*^	−0.002^*^	−0.126^***^	0.039^***^	42.1	35.1	6.03^***^
KBM	1220	0.657^***^	−0.001^***^	0.555^*^	−0.002^*^	−0.137^***^	0.260^***^	39.4	32.3	5.59^***^
EVF	1172	0.641^***^	−0.002^***^	0.548^*^	−0.002^*^	−0.147^***^	0.423^***^	43.0	36.1	6.26^***^
EVE	1175	0.707^***^	-0.002^***^	0.539^*^	−0.002^**^	−0.148^***^	0.220^***^	42.0	35.1	6.03^***^
Explained variable: Five-year EPS growth										
Q1	1028	0.815^***^	−0.0006	−0.035	5e-05	−0.196^***^	0.112^***^	45.3	38.5	6.65^***^
Q2	1045	0.470^***^	−0.001^*^	0.069	−0.0003	−0.131^***^	0.144^***^	46.0	39.5	7.08^***^
P/E	960	0.647^***^	0.001^*^	0.004	−0.164^***^	−0.129^***^	0.003^***^	45.2	38.2	6.49^***^
MV/BV	1034	0.894^***^	−0.0009	−0.067	0.0001	−0.199^***^	0.056^***^	45.8	39.0	6.80^***^
KBM	1067	1.068^***^	-0.0006	-0.045	7e-05	-0.231^***^	0.459^***^	46.2	39.7	7.10^***^
EVF	1028	1.113^***^	−0.0007	−0.056	0.0001	−0.256^***^	0.626^***^	46.2	39.5	6.88^***^
EVE	1026	1.139^***^	−0.0007	−0.075	0.0001	−0.240^***^	0.292^***^	46.2	39.5	6.87^***^
Explained variable: Ten-year EPS growth										
Q1	753	1.797^***^	−0.002	−9.885^**^	0.029^**^	−0.424^***^	0.192^***^	51.6	45.0	7.76^***^
Q2	766	1.462^***^	−0.003^**^	−10.41^**^	0.030^**^	−0.340^***^	0.208^***^	57.5	51.8	10.13^***^
P/E	699	1.507^***^	0.0005	−31.68^***^	−0.308^**^	−0.293^***^	0.005^**^	50.8	43.7	7.15^***^
MV/BV	759	2.010^***^	−0.003^***^	−11.41^**^	0.033^**^	−0.446^***^	0.098^***^	53.3	46.9	8.36^***^
KBM	782	2.502	-0.002^*^	-11.50^**^	0.033^**^	−0.557^***^	0.966^***^	54.4	48.3	9.03^***^
EVF	753	2.441^***^	−0.002	−10.68^**^	0.031^**^	−0.573^***^	1.256^***^	53.0	46.5	8.18^***^
EVE	752	2.676^***^	−0.003^**^	−11.77^***^	0.034^***^	−0.585^***^	0.669^***^	54.5	48.3	8.78^***^

Note: */**/*** The coefficients or F-statistic are significant at the 10% / 5% / 1% level

The main conclusions are in line with the findings for the FTSE100 companies. The incremental impact of the market level of growth opportunity for future earnings growth is confirmed for all growth opportunity measures, and EPS growth is lower in smaller companies, which is demonstrated by the negative and statistically significant coefficients with the firm size variable (lnMV_0_). Neither the effect of mean reversion nor earnings persistence was detected for the AIM companies – the coefficients at $RO{E}_{-1}$ and $\varDelta EP{S}_{0}$ vary in signs and statistical significance throughout the regressions; thus, we cannot draw any unequivocal conclusions. An increase in total assets (*ΔTA*_0_) has an impact on three-year and ten-year EPS growth that are slightly lower for the FTSE100 companies.

It can be concluded that growth opportunity measures are significantly related to the growth of value of companies as measured by EPS growth in the periods up to 10 years. The EPS growth is lower in smaller firms as measured by market value and we can conclude that mature companies with higher capitalization are value drivers on a capital market. The size of a company influence the EPS growth in a negative way on both markets but on a AIM market in a period of growth exceeding five years, growth of total assets influence the EPS growth in a positive way.

### Significance of the differences between the samples

The analyzis of the two groups of companies listed on LSE shows a strong relationship between growth opportunity measures, and their real growth. This finding confirms the expectations based on a theory.

In the next sted, we test the inequality of the correlation coefficients between the future growth measures and the growth opportunity measures in the two groups of companies according to the formula (9). The results are presented in Table 5.

**Table 5:** Testing the equality of correlation coeff. in the 2 groups of companies – results of the t-test

	gTA1	gTA3	gTA5	gTA8	gTA10
Q1	-3.956^***^	-3.616^***^	-3.840^***^	-2.827^***^	-3.737^***^
Q2	-2.071^**^	-3.158^***^	-1.802^*^	-1.534	-2.937^***^
P/E	-5.642^***^	-5.302^***^	-5.053^***^	-4.602^***^	-4.609^***^
MV/BV	-5.929^***^	-4.546^***^	-2.732^***^	-2.712^***^	-3.176^***^
KBM	-0.964	-3.718^***^	-3.379^***^	-1.604	-0.787
EVF	-2.816^***^	-2.247^**^	-2.120^**^	-1.260	-1.130
EVE	-2.475^**^	-1.773^*^	-1.244	-0.316	-0.686
	gS1	gS3	gS5	gS8	gS10
Q1	-2.857^***^	-2.702^***^	-4.142^***^	-2.689^***^	-3.159^***^
Q2	-1.925^*^	-2.393^**^	-1.402	-1.535	-1.614
P/E	-5.750^***^	-5.488^***^	-5.161^***^	-4.192^***^	-2.868^***^
MV/BV	-3.603^***^	-3.027^***^	-2.407^**^	-1.973^**^	-1.871^*^
KBM	-0.588	-2.134^**^	-2.088^**^	-0.611	-0.380
EVF	-1.825^*^	-1.647^*^	-1.701^*^	-0.371	0.723
EVE	-2.264^**^	-1.659^*^	-0.218	0.448	1.664^*^
	gE1	gE3	gE5	gE8	gE10
Q1	-4.682^***^	-4.769^***^	-3.056^***^	-4.008^***^	-3.669^***^
Q2	-3.437^***^	-5.253^***^	-2.844^***^	-3.558^***^	-4.758^***^
P/E	-5.571^***^	-4.668^***^	-2.410^**^	-3.049^***^	-3.383^***^
MV/BV	-5.200^***^	-4.472^***^	-3.386^***^	-2.705^***^	-2.288^**^
D/P	4.273	4.596^**^	3.884^**^	3.836	2.985
KBM	-0.064	-2.399^**^	-2.044^**^	-1.060	-0.171
EVF	-2.913^***^	-3.039^***^	-1.863^*^	-1.881^*^	-1.659^*^
EVE	-2.202^**^	-1.694^*^	-0.359	0.363	0.113
	gEPS1	gEPS3	gEPS5	gEPS8	gEPS10
Q1	0.788	2.355^**^	0.595	1.310	0.128
Q2	1.979^**^	2.395^**^	-0.126	0.528	0.460
P/E	0.251	-0.391	-1.224	-1.356	-1.126
MV/BV	-0.792	0.970	0.161	-0.499	-1.518
KBM	0.615	-0.218	-1.052	0.173	0.396
EVF	1.506	1.392	0.080	0.875	0.520
EVE	2.013^**^	1.637	0.067	1.271	0.675

Note: The table contains the values of the t-statistic. The null hypothesis states that the correlation coefficient between the growth opportunity measure and the realized future growth measure is the same in the two groups of companies. The alternative says these correlation coefficients are different

*/**/*** The null hypothesis can be rejected at the 10% / 5% / 1% level of statistical significance

According to the results presented in Table 5, about half of the correlation coefficients between the growth opportunity measures and the rates of future growth in total assets, sales, and equity differ in the two groups of companies at (at least) the 10% level of statistical significance. When it comes to correlation coefficients between the growth opportunity measures and future growth in earnings, the differences are negligible.

In the next step, we test the regression models that explain EPS growth rates for the difference between the two groups of companies according to the formulas (10) and (11). The results of both tests (12) are presented in the last two columns of Table 6.

**Table 6:** Determinants of future earnings growth in the joint group of companies from FTSE100 and AIM and the results of the Chow-test for structural change

	Sample	Const.	ROE_− 1_	ΔEPS_0_	ΔTA_0_	lnMV_0_	GO_0_	Adj. R^2^%	F-stat.	Chow- F-stat	LR-test chi^2^-stat
Explained variable: One-year EPS growth											
Q1	3370	0.033^***^	-1e-05	0.005	-2e-05	-0.006^***^	0.010^***^	4.7	34.1^***^	17.14^***^	101.6^***^
Q2	3388	0.036^***^	-4e-06	0.008	-2e-05	-0.005^***^	0.007^***^	3.1	22.9^***^	12.09^***^	72.0^***^
P/E	3279	0.041^***^	3e-07	-0.034	0.0003	-0.005^***^	0.0003^***^	2.9	20.5^***^	10.45^***^	62.3^***^
MV/BV	3376	0.047^***^	-1e-05	0.006	-2e-05	-0.006^***^	0.003^***^	3.9	28.7^***^	23.18^***^	136.7^***^
KBM	2179	-13.29^***^	2e-06	-0.042	0.0001	-0.004^***^	13.33^***^	1.6	8.02^***^	3.18^***^	19.0^***^
EVF	3370	0.047^***^	-1e-05	0.004	-1e-05	-0.006^***^	0.032^***^	3.8	27.90^***^	16.99^***^	100.7^***^
EVE	3371	0.054^***^	-7e-06	0.005	-2e-05	-0.007^***^	0.017^***^	3.4	25.16^***^	13.72^***^	81.5^***^
Explained variable: Three-year EPS growth											
Q1	3082	0.133^***^	-0.001^***^	1.233^***^	-0.004^***^	-0.019^***^	0.031^***^	9.9	68.97^***^	34.64^***^	201.9^***^
Q2	3108	0.131^***^	-0.001^***^	1.129^***^	-0.003^***^	-0.020^***^	0.037^***^	9.5	66.14^***^	40.00^***^	232.0^***^
P/E	3003	0.131^***^	3e-05	0.074	-0.0003	-0.017^***^	0.001^***^	5.3	34.37^***^	23.05^***^	135.7^***^
MV/BV	3087	0.167^***^	-0.001^***^	1.116^***^	-0.003^***^	-0.021^***^	0.016^***^	10.5	73.49^***^	42.39^***^	245.3^***^
KBM	1937	-40.54^***^	9e-05	0.074	-0.0002	-0.018^***^	40.71^***^	4.3	18.52^***^	15.98^***^	94.1^***^
EVF	3082	0.165^***^	-0.001^***^	1.222^***^	-0.003^***^	0.021^***^	0.130^***^	10.1	69.91^***^	44.18^***^	255.2^***^
EVE	3069	0.192^***^	-0.001^***^	1.208^***^	-0.004^***^	-0.023^***^	0.070^***^	9.0	61.65^***^	46.21^***^	266.4^***^
Explained variable: Five-year EPS growth											
Q1	2784	0.231^***^	-0.001^***^	0.222	-0.0007	-0.037^***^	0.068^***^	10.5	66.59^***^	48.25^***^	276.5^***^
Q2	2812	0.193^***^	-0.001^***^	0.247	-0.0008	-0.034^***^	0.073^***^	10.8	69.05^***^	59.23^***^	336.0^***^
P/E	2717	0.224^***^	0.0002	0.130	-0.0003	-0.030^***^	0.002^***^	5.6	33.51^***^	28.28^***^	165.3^***^
MV/BV	2790	0.304^***^	-0.002^***^	0.133	-0.0005	-0.040^***^	0.025^***^	9.5	59.49^***^	45.53^***^	261.7^***^
KBM	1709	-83.32^***^	0.0004^**^	0.152	-0.0004	-0.035^***^	83.62^***^	5.9	22.33^***^	23.93^***^	138.8^***^
EVF	2784	0.314^***^	-0.001^***^	0.221	-0.0007	-0.041^***^	0.225^***^	9.5	59.51^***^	52.01^***^	297.0^***^
EVE	2759	0.350^***^	-0.001^***^	0.224	-0.0007	-0.044^***^	0.117^***^	8.5	52.20^***^	46.17^***^	265.1^***^
Explained variable: Ten-year EPS growth											
Q1	2131	0.489^***^	-0.002^***^	2.245	-0.007	-0.087^***^	0.161^***^	11.5	56.49^***^	39.41^***^	225.4^***^
Q2	2156	0.498^***^	-0.002^***^	1.882	-0.006	-0.091^***^	0.182^***^	11.6	57.47^***^	57.69^***^	322.7^***^
P/E	2088	0.481^***^	0.0004	3.783	-0.001	-0.063^***^	0.003^***^	4.8	22.00^***^	15.88^***^	93.7^***^
MV/BV	2141	0.670^***^	-0.004^***^	0.766	-0.002	-0.093^***^	0.063^***^	11.5	56.71^***^	46.89^***^	265.7^***^
KBM	1204	-127.6^***^	0.001^***^	4.684^**^	-0.014^**^	-0.070^***^	128.21^***^	6.6	17.89^***^	25.94^***^	147.8^***^
EVF	2131	0.704^***^	-0.001^***^	2.243	-0.006	-0.098^***^	0.493^***^	9.8	47.43^***^	39.37^***^	225.2^***^
EVE	2109	0.833^***^	-0.001^**^	2.534	-0.007	-0.110^***^	0.263^***^	9.5	45.18^***^	39.34^***^	224.9^***^

Note: The table contains the estimates of the regression models in the joint group of companies. The results of two tests for a structural break in the regression coefficients when the companies are divided into FTSE and AIM are presented in the last two columns. The null hypothesis in both tests states that the regression coefficients are stable in the whole group of companies

*/**/*** The null hypothesis can be rejected at the 10% / 5% / 1% level of statistical significance

The results of both tests are the same. In all regressions, the coefficients are not stable when moving from the FTSE100 companies to the AIM companies. These results indicate that the structural relationships between future EPS growth and the different factors that predict this growth (including the growth opportunity measures) are significantly different in both groups of companies, and separate regression models for surveyed groups of companies should be estimated.

## Discussion of the results

When the FTSE100 companies were analyzed, almost all growth opportunity measures performed very well in every time horizon. When the regression models with fixed effects were taken into consideration, the conclusions about the statistical importance of the incremental impact of growth opportunity measures were evident. The results of the presented research do not confirm the DHJ findings, who also studied the UK market and found no relationship between growth opportunity measures and growth of EPS calling this phenomena a “puzzle”.

EPS growth was also related to firm size and for mature companies growth of EPS was negatively influenced by a market size and a book market growth in all periods. In smaller companies in periods exceeding five years, the growth of total assets influenced the EPS growth in a positive way.

The differences between the analyzed markets were examined to identify how companies behaved depending on the market where they are listed. In all regressions, the coefficients were not stable when moving from the FTSE100 companies to the AIM companies. These results indicate that the structural relationships between future EPS growth and different factors that predict this growth were significantly different in both groups of companies.

The EPS growth of companies traded on different markets (main or alternative) is characterized by different regression models, which represent different growth patterns related to the stage of development. Similar conclusions were presented by Bolek et al. (2021) for the Warsaw Stock Exchange in Poland. Thus, it can be concluded that the expected growth is related to future growth within a given market segment. This finding indicates that EPS growth process is specific and related to companies’ different stages of development and different investors’ expectations.

## Conclusion

The purpose of this study was to analyze the relationship between the measures of expected growth and the future growth of companies listed on the London Stock Exchange.

In this research paper the following hypothesis are tested: (1) the growth opportunity ratios are significantly related to the future real growth of enterprises listed on LSE and (2) there are significant differences between tested samples and they may explain earlier failures in finding the relationship between growth opportunity and the real growth of companies as presented by DHJ.

The theoretical assumptions indicate a relationship between growth opportunity and real growth of companies, however, previous studies of the UK market did not confirm it unequivocally. The relationship between companies’ growth opportunities and real growth was previously confirmed by the authors in a study of the Polish markete.The motivation of this paper is related to the application of the method proposed by Bolek et al. (2021) who studied WSE listed companies divided for a mature firms included in a WIG index and less mature firms included in NewConnect index, on a more developed UK market and solve the problem of market inefficiency reported by DHJ who stated that growth opportunity measures calculated based on a market price are not related to the the real growth of companies as measured by EPS growth. According to the presented results the relationship between growth opportunity measures and real growth of companies for a developed market such as the UK indicates that the research within a given sub-market (companies included in FTSE100 and AIM all-shares indices) causes the results to be statistically significant and in line with theory. The relationship between growth and growth opportunity measures is significant in the presented study because the samples differ and should be surveyed separately.

The presented research results can be used by investors in the context of another dimension of market efficiency, where measures of growth opportunity should reflect the growth of enterprises as measured by EPS. In the future research the explanation of the differences between markets will be explored and efficient market hypothesis will be developed based on growth potential and EPS growth relationship.

## Data Availability

The datasets generated during and/or analysed during the current study are available from the corresponding author on reasonable request.

## Footnotes

1 “Reports: Issuer List, May 2021” (xlsx). London Stock Exchange. May 2021.
